# Comparison of serological methods with PCR-based methods for the diagnosis of community-acquired pneumonia caused by atypical bacteria

**DOI:** 10.1186/s12952-016-0047-y

**Published:** 2016-03-02

**Authors:** Mariana Herrera, Yudy Alexandra Aguilar, Zulma Vanessa Rueda, Carlos Muskus, Lázaro Agustín Vélez

**Affiliations:** Grupo Investigador de Problemas en Enfermedades Infecciosas (GRIPE), Sede de Investigación Universitaria, Calle 62 # 52-59, Laboratorio 630, Universidad de Antioquia, Medellín, Colombia; Corporación de Ciencias Básicas Biomédicas, Universidad de Antioquia UdeA, Medellín, Colombia; Universidad Pontificia Bolivariana, Medellín, Colombia; Programa de Estudio y Control de Enfermedades Tropicales (PECET), Universidad de Antioquia UdeA, Medellín, Colombia; Infectious Disease Section, School of Medicine, Universidad de Antioquia UdeA, Medellín, Colombia

**Keywords:** *M. pneumoniae*, *L. pneumophila*, *C. pneumoniae*, Multiplex PCR, Atypical pneumonia, Molecular diagnosis

## Abstract

**Background:**

The diagnosis of community-acquired pneumonia (CAP) caused by *Legionella pneumophila, Mycoplasma pneumoniae*, and *Chlamydophila pneumoniae* is traditionally based on cultures and serology, which have special requirements, are time-consuming, and offer delayed results that limit their clinical usefulness of these techniques. We sought to develop a multiplex PCR (mPCR) method to diagnosis these bacterial infections in CAP patients and to compare the diagnostic yields obtained from mPCR of nasopharyngeal aspirates (NPAs), nasopharyngeal swabs (NPSs), and induced sputum (IS) with those obtained with specifc PCR commercial kits, paired serology, and urinary antigen.

**Results:**

A total of 225 persons were included. Of these, 10 patients showed serological evidence of *L. pneumophila* infection, 30 of *M. pneumoniae*, and 18 of *C. pneumoniae*; 20 individuals showed no CAP. The sensitivities were mPCR-NPS = 23.1 %, mPCR-IS = 57.1 %, Seeplex®-IS = 52.4 %, and Speed-oligo®-NPA/NPS = 11.1 %, and the specificities were mPCR-NPS = 97.1 %, mPCR-IS = 77.8 %, Seeplex®-IS = 92.6 %, and Speed-oligo®-NPA/NPS = 96.1 %. The concordance between tests was poor (kappa <0.4), except for the concordance between mPCR and the commercial kit in IS (0.67). In individuals with no evidence of CAP, positive reactions were observed in paired serology and in all PCRs.

**Conclusions:**

All PCRs had good specificity but low sensitivity in nasopharyngeal samples. The sensitivity of mPCR and Seeplex® in IS was approximately 60 %; thus, better diagnostic techniques for these three bacteria are required.

**Electronic supplementary material:**

The online version of this article (doi:10.1186/s12952-016-0047-y) contains supplementary material, which is available to authorized users.

## Background

Infections by the atypical bacteria *Mycoplasma pneumoniae, Chlamydophila pneumoniae,* and *Legionella pneumophila* are frequent causes of community-acquired pneumonia (CAP) in both children and adults [1–3]. Latin America has reported CAP figures caused by these bacteria ranging from 1.7 to 15.7 % for *M. pneumoniae*, 3.4 to 6.1 % for *C. pneumoniae,* and 1.1 to 4 % for *L. pneumophila* [3, 4].

Diagnosis of these bacteria is traditionally based on cultures and serology, which involve special technical requirements that are costly and time-consuming, offer delayed results, and in the case of serology, require a second convalescent-phase sample, which limits the clinical usefulness of these techniques [5–7]. This explains why although the circulation of atypical bacteria in the region is evident, these bacteria can only be diagnosed in very specialized reference centers. Due to this aspect, and because the clinical presentation does not differ significantly from that caused by pyogenic bacteria or respiratory viruses [8], the perception is that these agents are rare in these countries. The therapeutic consequence of this omission is the prescription of insufficient treatments in some cases or treatments that are excessive and unnecessary in others.

Given these problems, nucleic acid amplification techniques are often used, including conventional PCR, real-time PCR (qPCR), and in-house or commercial mPCR [9–11]. These are considered faster, more sensitive, and more specific than cultures and serology [12]. However, the possibility of contamination and the difficulties of interpreting positive cases as disease or colonization are the main limitations. Although several commercial kits for the detection of *M. pneumoniae, C. pneumoniae*, and *L. pneumophila* are now available [10, 13–15], limited information is available in the literature regarding the validation process of such tests. The existing studies have limited information about the clinical condition of the study population in which the tests were validated, the samples used, and the molecular targets; some studies compared only the commercial kit with another in-house or commercial molecular test, without using any other accepted reference tests (culture or paired serology). Additional file 1 describes the heterogeneity of the previously conducted studies.

To investigate a possible solution to these diagnostic difficulties, our aim was to standardize and validate an in-house mPCR for a quick and timely diagnosis of CAP caused by these atypical bacteria in a single reaction. In addition, we sought to evaluate the diagnostic performance of mPCR in different respiratory samples, namely, nasopharyngeal aspirates (NPAs), nasopharyngeal swabs (NPSs) and induced sputum (ISs), and to compare this performance with that of existing PCR commercial kits, paired serology, and urinary antigen.

## Results

### Standardization of multiplex PCR

The primers used allowed the amplification of the gene fragments of interest: *mip* from *L. pneumophila*, *pst*I from *C. pneumoniae* and *p*1 from *M. pneumonia*e, and these primers showed no cross-reactions among the bacteria, either with related species or other microorganisms, according to the specificity analysis of the reaction obtained with the BLAST program. The conditions, under which optimal mPCR amplification was achieved in a final volume of 25 μL, were 0.05 U/μL Taq polymerase (Fermentas St. Leon-Rot, Germany), 1X Taq buffer with KCl, 2.0 mM MgCl_2_, 0.2 mM dNTPs, 0.3 μM concentrations of each primer, 0.1 mg/μL BSA, and 6 μL of DNA (The median concentration of the extracted DNA from each sample was 4.9 ng/μL, upper limit: 166.18 ng/μL, lower limit: 2.11 ng/μL). The cycling conditions in the C1000 thermal cycler (BioRad, CA, USA) were as follows: one cycle of DNA denaturation at 95 °C for 5 min; 35 cycles of denaturation at 94 °C for 45 s, primer annealing at 58 °C for 60 s and primer extension at 72 °C for 45 s; and a final extension at 72 °C for 7 min.

**Fig. 1:**
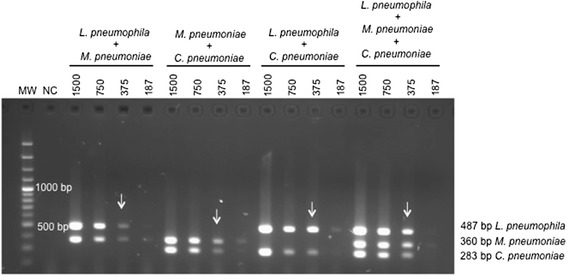
Analytical sensitivity of mPCR using 1,500; 750; 375; and 187 copies of *L. pneumophila mip* genes, *p*1 of *M. pneumoniae*, and *Pst*l of *C. pneumoniae* MW: 100 bp molecular weight marker; NC: negative control; Lines marked with arrows correspond to the amplicons from 375 copies of each gene

**Fig. 2:**
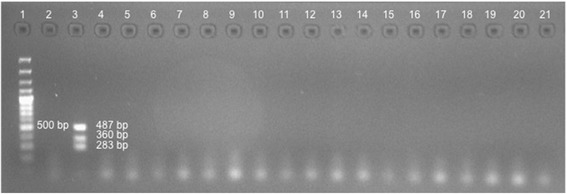
Analytical specificity of mPCR. **1**. Molecular weight marker 100 bp; **2**. Negative control; **3**. Positive control (487 bp *L. pneumophila,* 360 bp *M. pneumoniae,* and 283 bp *C. pneumoniae*); Bacteria: **4**. *Streptococcus pneumoniae;* **5**. *Haemophilus influenzae;* **6**. *Klebsiella pneumoniae;* **7**. *Escherichia coli;* **8**. *Pseudomonas aeruginosa;* **9**. *Staphylococcus aureus;* **10** *. Nocardia* spp.; **11**. *Enterobacter cloacae*; Fungi: **12**. *Histoplasma capsulatum*; **13**. *Aspergillus terreus*; **14**. *Cryptococcus neoformans*; **15**. *Candida tropicalis*; **16**. *Candida albicans*; **17**. *Candida guilliermondii*; **18**. *Candida glabrata*; **19**. *Paracoccidioides brasiliensis*; **20**. *Mycobacterium tuberculosis* (bacteria); **21**. Human DNA

Standardized PCR had a detection limit of 375 copies for each gene, regardless of whether the PCR was set-up to amplify a single gene or two or three genes simultaneously (Fig. 1); however, some amplification was observed with 187 copies of DNA, especially when a DNA mixture of two bacterial strains was run. No cross-amplification with DNA from the 17 different pathogens and/or colonizing microorganisms of the respiratory tract or with human DNA (Fig. 2) was observed.

Standardized mPCR was reproducible using a concentration of 750 copies of each gene when six PCR reactions were run simultaneously (intra-assay reproducibility) and on six different days (interassay reproducibility). Regardless of the test day, the intensity of the signal did not vary.

### Clinical and epidemiological characteristics

A total of 205 individuals with CAP were analyzed in three groups – 68 adults in Group 1, 88 adults in Group 2 and 49 children in Group 3. Table 1 describes the main characteristics of these three groups. The etiology observed in Table 1 does not reflect the percentage distribution of the microorganisms found in the evaluated cohorts but is due to the selection of patients required to evaluate the techniques being studied.

**Table 1:** Clinical and epidemiological characteristics of the population with CAP

Variables	Group 1 (*n* = 68)	Group 2 (*n* = 88)	Group 3 (*n* = 49)
Age in years, Median (Q_1_-Q_3_)	65 (41–76)	63 (40–76)	3 (1–7)
Males, n (%)	28 (41.2)	49 (55.7)	29 (59.2)
Received antibiotics in last 3 months, n (%)	19 (27.9)	10 (11.4)	9 (18.4)
Symptom duration, in days, Median (Q_1_-Q_3_)	7 (4–15)	6 (3–10)	4 (2–8)
Presence of comorbidities, n (%)	37 (54.4)	50 (56.8)	2 (4.1)
Chronic obstructive pulmonary disease	24 (35.3)	37 (42)	0
History of convulsions in the last month	0	3 (3.4)	2 (4.1)
Severe pneumonia^a^, n (%)	15 (22.1)	26 (29.5)	1 (2.0)
Frequency of atypical bacteria, n (%)			
*M. pneumoniae*	5 (10.9)	4 (5.2)	21 (43.8)
*C. pneumoniae*	3 (6.5)	9 (11.7)	6 (12.5)
*L. pneumophila*	3 (6.5)	2 (2.6)	5 (10.4)
Length of hospital stay, in days, Median (Q_1_-Q_3_)	6 (3–9)	7 (4–10)	4 (2–9)
In-hospital death, n (%)	0	8 (9.1)	0

^a^In children (group 3), this corresponds to the WHO classification of very severe pneumonia

Most of the 20 individuals in group 4 (control group) were male (60 %) and had a median age of 50 years (Q1 to Q3 = 29 to 55). Eight of the 10 individuals who suffered rheumatic diseases had been diagnosed with rheumatoid arthritis; 1, with systemic lupus erythematosus; and 1, with Sjögren’s syndrome. Three of them were receiving tumor necrosis factor-alpha (anti-TNFα) antagonists at the time of inclusion in the study.

### Test results

Among the 225 patients included in the 4 groups, 190 paired serologies were performed (46 in group 1, 77 in group 2, 48 in group 3, and 19 in group 4). In addition, 88 mPCR were performed in NPAs, 137 mPCR in NPSs, 49 mPCR and 49 Seeplex® Pneumobacter in IS, and 161 Speed-Oligo® in NPAs or NPSs. The *L. pneumophila* urinary antigen was positive in only one patient in group 2, who also exhibited a positive paired serology; because of that, this urinary antigen was not considered as a gold standard.

The results of the negative and positive controls of the serology tests, the urinary antigen and the different molecular tests were always negative and positive, respectively. The inhibition control of the PCRs was positive in all samples tested, indicating the absence of PCR inhibitors.

In samples obtained from hospitalized patients showing CAP symptoms and distributed among groups 1, 2 and 3, mPCR was only positive for *M. pneumoniae* in one sample in group 1 and in 25 samples of group 3 (7 samples of NSP and 18 IS samples). No amplification was observed for *C. pneumoniae* and *L. pneumophila* in any of the samples with mPCR. In contrast, with commercial PCR (Speed-oligo or Seeplex), amplification was achieved in a larger number of samples undergoing mPCR. With commercial PCR, a total of 18 *M. pneumoniae-*positive samples were detected in the three groups (4 in group 1, 1 in group 2, and 13 in the group 3). Only 1 sample in group 2 was positive for *C. pneumoniae,* and 2 samples in group 1 were positive for *L. pneumophila* (Table 2).

**Table 2:** Positive results of serology, in-house mPCR, and commercial PCR classified by atypical bacteria

Techniques	Group 1^a^ NPS	Group 2^b^ NPA	Group 3^c^ NPS and IS	Group 4^d^ NPS
*M. pneumoniae*	n (%)	n (%)	n (%)	n (%)
Serology	5/46 (10.9)	4/77 (5.2)	21/48 (43.8)	2/19 (10.5)
mPCR	1/68 (1.5)	0/88 (0)	NPS: 7 (14.3) IS: 18 (36.7)	0/20 (0)
Commercial PCR [Trade mark]	4/68 (5.9) [Speed-oligo]	1/88 (1.4) [Speed-oligo]	13/49 (26.5) [Seeplex]	0/20 (0) [Speed-oligo]
*C. pneumoniae*				
Serology	3/46 (6.5)	9/77 (11.7)	6/48 (12.5)	4/19 (21.1)
mPCR	0/68 (0)	0/88 (0)	NPS: 0 (0) IS: 0 (0)	0/20 (0)
Commercial PCR	0/68 (0)	1/88 (1.4)	0/49 (0)	0/20 (0)
*L. pneumophila*				
Serology	3/46 (6.5)	2/77 (2.6)	5/48 (10.4)	0/19 (0)
mPCR	0/68 (0)	0/88 (0)	NPS: 0 (0) IS: 0 (0)	1/20 (5.0)
Commercial PCR	2/68 (2.9)	0/88 (0)	0/49 (0)	4/20 (21)

*NPS* Nasopharyngeal swab, *NPA* Nasopharyngeal aspirate, *IS* Induced sputum

^a^Prospective adults with community-acquired pneumonia (CAP). Speed-Oligo® was run as commercial PCR on the NPSs

^b^Retrospective adults with CAP. Speed-Oligo® was run as commercial PCR on the NPAs

^c^Children with CAP. Seeplex® PneumoBacter was run as a commercial PCR on IS

^d^Individuals without CAP. Speed-oligo® was run as a commercial PCR on NPSs

When assessing the positivity of serology by quadrupling the antibody titers, the technique considered the gold standard in this study, serology was observed to detect a greater number of positive samples than any of the 3 types of PCR used in this study. For *M. pneumonia*, 30 samples were positive (5 in group 1, 4 in the group 2, and 21 in the group 3). For *C. pneumoniae*, 18 samples were positive (3 in group 1, 9 in group 2, and 6 in group 3), whereas 10 samples were positive for *L. pneumophila*, (3 in group 1, 2 in group 2, and 5 in group 3). In group 3, two types of samples (NPSs and IS) were evaluated by mPCR. Only the presence of DNA from *M. pneumonia* was detected in 25 samples. Of these, 18 samples were positive for the IS, and 7, for the NPSs.

Interestingly, in samples from the control group and without symptoms of CAP, 6 serologically positive samples were detected. Of these, 2 were positive for *M. pneumoniae*, and 4, for *C. pneumoniae*. In addition, one sample was positive for mPCR, and 4, for commercial PCR for *L. pneumophila*.

Finally, when analyzing the samples obtained from individuals with CAP in a global and comprehensive manner, that is, without division by groups, *M. pneumoniae* was the most detected bacteria by any of the three methods. By serology, 30 samples were detected, and by mPCR, 26 samples, whereas 18 samples were positive by cPCR. For *C. pneumoniae,* only one sample was positive by cPCR, and 18 were positive by serology, whereas for *L. pneumophila,* 2 samples were positives by cPCR, and 10, by serology (Table 2).

Given that no positive cases of *C. pneumoniae* and *L. pneumophila* were obtained by mPCR, and very few cases, by commercial PCR, only the operating characteristics of the PCRs to *M. pneumoniae* are presented below.

Table 3 shows that the PCRs exhibit high specificity with low sensitivity in the nasopharyngeal samples for both the NPAs and NPSs. The sensitivity was higher in the IS, but was only 57.1 % for mPCR and 52.4 % for Seeplex® PneumoBacter. In turn, when the PCRs with the highest sensitivity in IS were compared with each other, PCR Seeplex® PneumoBacter exhibited higher specificity and positive predictive value than mPCR.

**Table 3:** Operational features of the PCRs used for *M. pneumoniae*

Test (N)	Sensitivity (%)	Specificity (%)	PPV (%)	NPV (%)
mPCR NPA (77)	N/A	N/A	N/A	N/A
mPCR NPS (94)	23.1 (4.9–41.2)	97.1 (92.3–100)	75 (38.7–100)	76.7 (67.2–86.2)
mPCR IS (48)	57.1 (33.6–80.7)	77.8 (60.2–95.3)	66.7 (42.1–91.2)	70 (51.9–88.1)
Seeplex® PneumoBacter IS (48)	52.4 (28.6–76.1)	92.6 (80.7–100)	84.6 (61.2–100)	71.4 (55.0–87.8)
Speed-oligo® NPA/NPS (111)	11.1 (0–37.2)	96.1 (91.8–100)	20 (0–65.1)	92.4 (86.9–97.9)

Values calculated with paired serologists as the gold standard. *NPA* Nasopharyngeal aspirate. *NPS* Nasopharyngeal swab. *IS* Induced sputum. *PPV* Positive predictive value. *NPV* Negative predictive value. *N/A* Not applicable (these values cannot be calculated for this test due to the lack of positive results in serology that coincide with the positive results in mPCR of NPAs)

**Fig. 3:**
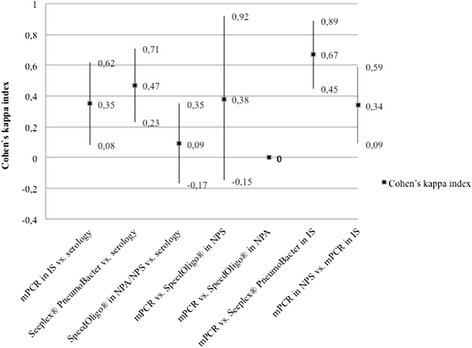
Concordance (kappa index) between in-house (mPCR) and commercial PCR for *Mycoplasma pneumoniae.* NPS: Nasopharyngeal swab; NPA: Nasopharyngeal aspirate; IS: Induced sputum

Under our PCR conditions, the concordance between methods in a single sample and between samples with a single method was very low (kappa coefficient <0.4). Interestingly, in the IS, the concordance was better between mPCR and Seeplex (kappa = 0.67) (Fig. 3).

## Discussion

The results of this study allow us to highlight three key aspects: 1) the in-house PCR standardized in this study and the commercial PCRs used had low sensitivity and poor concordance compared with the paired serology; 2) the IS sample had the best performance for the diagnosis of *M. pneumoniae* by PCR compared with those obtained by NPAs and NPSs; and 3) the quadrupling of titers in the paired serology for *C. pneumoniae* and *M. pneumoniae* could occur in individuals without CAP. Among them, PCRs could also be positive for *L. pneumophila*.

Regarding the first point, the literature has reported good agreement between the in-house and commercial PCRs performed on sputum, bronchoalveolar lavage (BAL), and endotracheal aspirates with paired serology results [16]; however, in our study, the concordance between the evaluated PCRs and serology was very low. Templeton et al. reported similar findings in 2003; they found that out of 106 samples tested, 12 were positive by 3 methodologies other than PCR, but only 8 of these were positive by paired serology [17]. The finding of positive PCRs in respiratory secretions without quadrupling in antibody titers in patients with CAP may occur because these patients are asymptomatic carriers of *M. pneumoniae* or *C. pneumoniae* in the respiratory epithelium or because of the persistence of these bacteria or their nucleic acids in the respiratory tract following previous infections [18–20]. Similarly, the false negative results of PCRs could be explained by a bacterial load below the detection limit of the PCRs, previous antibiotic treatment in patients, dilutions of samples when added to the transport medium, degradation of significant amounts of DNA during the sample storage process, or the presence of interfering DNA coming from human cells or other colonizing microorganisms of the respiratory tract, which would affect amplification. The positive and negative results in the controls in all experiments (serology, urinary antigen, and molecular techniques) ruled out the possibility of experimental error, and the amplification of the inhibition controls ruled out the presence of PCR inhibitors.

With regard to the sensitivity obtained for in-house mPCR and commercial kits using the quadrupling of titers in paired serology as the gold standard, this was lower than previous studies [15, 21], in which the sensitivity ranged from 66.7 to 97.3 %. Even sensitivities and specificities of up to 100 % have been described when the gold standard used was a PCR monoplex assay and the study population was comprised solely of positive individuals confirmed by this technique [22] (53). This feature highlights the importance of knowing the characteristics of the study population, the type of respiratory sample being used (sputum, NPS, NPA, or BAL) and the inhibitors potentially present in each of them, the population where the PCR (adults, children, or elderly) is being assessed, and the various molecular targets being used.

NPSs and NPAs have been proposed as good choices of sample type for the diagnosis of CAP when resorting to non-invasive samples [23], but for the diagnosis of atypical bacteria, sputum has a higher performance than NPSs [24, 25], and in turn, these samples are superior to NPAs [26]. The results of our study were consistent with this claim; that is, we found that the PCR results varied for the diagnosis of *M. pneumoniae* depending on the type of respiratory specimen used, as the IS enabled the identification of a greater number of cases. In this regard, Collier and Clyde [27] and Kenny et al. [28] indicated that sputum samples were superior for the detection of *M. pneumoniae* because the number of bacteria are higher in the pulmonary alveolus than in the epithelium of the upper respiratory tract of patients with pneumonia. However, Reznikov et al. [26] reported that the PCR for *M. pneumoniae* in NPAs and NPSs had similar positivity percentages (45 and 50 %, respectively) but a greater presence of inhibitors in NPAs (36 %) than in NPSs (0 %).

The type of population also affects the operational features of the PCRs in that the results of paired serology vary according to patient age, prior exposure to these bacteria, or the presence of comorbidities. Acute *M. pneumoniae* infections in children are characterized by significant increases in IgM antibodies but can only increase titers of IgG or both immunoglobulins; also, the IgM titers may remain high for several months or even years [29], which constitutes the main limitation of this test. However, adults can respond by increasing only IgG, especially when a re-infection occurs by this germ, or they may be unable to mount an appropriate serological response due to deficiencies in the immune system, which are common in patients of certain ages [30] or with underlying diseases. Examples include immunocompromised individuals or those with rheumatologic diseases [31]. Therefore, the gold standard against which these molecular diagnostic techniques are being assessed is far from being the ideal test. Furthermore, PCR detection of atypical bacteria also has limitations; no consensus exists regarding which molecular target should be amplified to achieve higher sensitivity and specificity, nor does a clearly defined standard protocol exist [21, 32–34]. Depending on the selected molecular target, in which one or multiple copies could be in the investigated genome, the amount of DNA of the microorganisms present in the sample can vary significantly. Even when the presence of the same gene is studied using two different molecular tests, such as Speed-oligo® and mPCR, the results may show poor consistency. This may be due to differences in the methodologies used (including the type of PCR -- monoplex versus multiplex -- and the revealing technique -- oligochromatography versus agarose gel electrophoresis) or because of the amplification of the different regions of the same gene [16]. Although some authors reported similar results when they used a single PCR or a duplex assay to detect two of these pathogens [14], others argue that the conventional format for some PCRs is more sensitive than the multiplex [35], which may have contributed, at least in part, to some false negatives obtained with mPCR in our study.

Finally, the positive results obtained by serology and PCR in individuals without CAP require a better definition of the role of the causative microorganisms in the respiratory microbiome of these subjects and of the usefulness of this serological test as the gold standard. Both *M. pneumoniae* and *C. pneumoniae* are bacteria that are known to colonize the respiratory tract [18–20]. Recent studies show the presence of *M. pneumoniae* and *C. pneumoniae* in asymptomatic individuals (by culture, serology, or detection of DNA). Therefore, detection of these pathogens by PCR does not necessarily indicate disease, and such studies make it clear that none of the methods currently used for diagnosis make it possible to differentiate the carrier state of symptomatic infection [18–20]. It is possible that because many infections caused by these pathogens are asymptomatic, some of the patients without CAP who served as controls may have been recently infected by the pathogens without developing the disease [36], which potentially helps explain the serology conversions observed in these individuals.

In addition, in 2010, Villegas et al. claimed that *C. pneumoniae* serology can give false positives due to cross-reactions in cases of acute infection because of the presence of heterotypic antibodies [37]. A similar phenomenon can be observed with *M. pneumoniae*, whose acute infections are often characterized by the transient generation of autoantibodies, which are considered responsible for many of their extrapulmonary manifestations, and, as shown by our results, some patients with autoimmune diseases may yield false positive results.

In this study, Speed-oligo® for *L. pneumophila* was positive in four patients with rheumatic diseases, whereas mPCR was positive in one of those cases. Although the carrier state for this germ has not been described, several possible explanations exist for this finding. Either people were colonized or were at risk of becoming ill because of the bacteria [38, 39], or these results were false positives of the PCRs, results that cannot be attributed to cross-contamination with other samples as the extraction controls and amplification of PCRs were always negative.

One limitation of the study was the absence of cultures as a gold standard for diagnosis, particularly because such cultures may help to clear up discordant cases. Another possible limitation was that to complete the sample size, we had to resort to various groups of patients (adults and children admitted prospectively and retrospectively). Although these groups were analyzed separately and we were able to evaluate how the tests behaved among themselves in different samples and different populations, the sample size per group was low. Further studies that prospectively evaluate these aspects are required.

## Conclusions

This study demonstrates that the molecular tests (in-house and commercial) and the reference tests evaluated for the diagnosis of atypical bacteria in patients with CAP have low sensitivity, and do not allow discrimination between those patients with acute or convalescent infection and asymptomatic carriers. Thus, the development of better techniques is needed for the diagnosis of CAP caused by *M. pneumoniae*, *C. pneumoniae*, and *L. pneumophila*. Such studies should include prospective evaluations of different sample types and molecular targets, quantification of bacterial DNA, pediatric populations and healthy adults, individuals with suspected CAP infection by these microorganisms, immunocompetent and immunocompromised individuals, and different molecular techniques.

## Methods

### Standardization of mPCR

DNA from *M. pneumoniae* strain FH of Eaton Agent (gene *p1*), *C. pneumoniae* strain CM-1 (gene *PstI*), and *L. pneumophila* strain Philadelphia-1 (gene *mip*) from the American Type Culture Collection (ATCC® Virginia, USA) was used for the standardization of mPCR, according to the protocol and primers described by McDonough et al. [40] (Additional file 2). The specificity of the primers was verified using the BLAST program, and the tendency to form homo- and heterodimers, in addition to secondary structures, was evaluated using the Oligo Analyzer program (IDT Technologies, www.idtdna.com/calc/analyzer). The optimal concentrations of the PCR reagents were experimentally determined: primers (0.2–1.0 μM), Taq polymerase (0.05–0.3U/μL), Magnesium chloride (1.0–2.5 mM), and bovine serum albumin (BSA (0.1–0.7 μg/μL) as adjuvant. The best annealing temperature was selected by performing a temperature gradient between 55 °C and 66 °C; in addition, primer annealing and extension were evaluated between 30 and 60 s. The optimal conditions were selected according to the points to achieve the sharpness of banding with the lowest DNA concentration.

PCR reactions were revealed using 2 % agarose gel electrophoresis (AMRESCO®, USA), stained with EZ-VISION™ (AMRESCO®, USA); the gel was run at 70 V for 50 min. Gel images were obtained using the ChemiDoc XRS (BioRad) equipment and the Quantity One® program.

### Determination of analytical sensitivity and specificity

The determination of analytical sensitivity was performed using serial dilutions of DNA from the strains obtained from ATCC or with plasmids containing gene-specific inserts. Each amplified fragment was ligated to the pGEM®-Teasy plasmid (Promega®, Southampton, USA) according to the manufacturer’s instructions. Then, the recombinant plasmids were purified using the Wizard® plus SV Miniprep DNA Purification System (Promega®, Southampton, USA), linearized and quantified using NanoDrop®. The number of copies was calculated from the obtained nanograms [41, 42], and serial dilutions were made. Analytical specificity was evaluated with DNA from different sources at a concentration of 4 ng/μL. We evaluated human DNA from peripheral blood cells and DNA from pathogenic and frequent colonizers of the respiratory tract. These colonizers included the bacteria *Streptococcus pneumoniae, Staphylococcus aureus, Haemophilus influenzae, Escherichia coli, Enterobacter cloacae, Mycobacterium tuberculosis, Nocardia* spp., *Klebsiella pneumoniae* ATCC10031 and *Pseudomonas aeruginosa* ATCC PA01 and the fungi *Candida albicans, Candida tropicalis, Candida guilliermondii, Candida glabrata, Histoplasma capsulatum, Cryptococcus neoformans, Aspergillus terreus*, and *Paracoccidioides brasiliensis*. A dilution corresponding to 750 copies of each gene was used to evaluate reproducibility. Furthermore, mPCR was run six times in one day to determine intra-assay reproducibility and on six different days to verify inter-assay reproducibility.

### Validation of multiplex PCR

To validate the mPCR technique, a sample size of 188 patients with CAP was calculated, taking into account an expected sensitivity of 92 % for mPCR, a prevalence of CAP in the city caused by these three atypical bacteria of 24.4 %, and a confidence level of 92 %. All patients had to be hospitalized.

### Study population

The population consisted of four study groups; the first three groups involved patients hospitalized with CAP who were not severely immunosuppressed. Group 1 consisted of 68 patients who were prospectively enrolled for this study, whereas the patients in group 2 (*n* = 88) and 3 (*n* = 49) were taken from two previous studies conducted by our group. Positive cases were selected by quadrupling the titers for these atypical bacteria, and the patients with CAP caused by other pathogens or of unknown etiology were selected randomly until the estimated sample size was attained. The fourth group included individuals without pneumonia (controls) and was divided into two subgroups of equal numbers of patients. One subgroup consisted of blood donors who were completely healthy; the other included patients with rheumatic diseases who were at a higher risk of false positive reactions in paired serology (Table 4).

**Table 4:** Eligibility criteria of the study population

	Group 1 (*n* = 68)	Group 2 (*n* = 88)	Group 3 (*n* = 49)	Group 4 (*n* = 20)
Recruitment	Prospective, recruited from 2010 to 2012	Retrospective, recruited from 2005 to 2006.	Prospective, recruited from 2011 to 2012	Prospective, recruited from 2011 to 2012
Inclusion Criteria	1. Adults ≥18 years 2. Hospitalized with CAP, with radiologic evidence 3. Agreed to participate in the study 4. Available NPS, urine, and blood samples	1. Patients ≥18 years 2. Hospitalized with CAP, with radiologic evidence 3. Agreed to participate in the study	1. Children between 1 month and 17 years 2. Hospitalized with CAP, with radiologic evidence 3. Agreed to participate in the study 4. Under 15 days of symptoms	1. Adults ≥18 years 2. Agreed to participate in the study 3. Completely healthy, recruited from blood banks, or individuals with rheumatic conditions, without respiratory infection, who could be receiving tumor necrosis factor antagonists (anti-TNF)
Exclusion Criteria	1. Hospitalization during the 2 weeks prior to recruitment 2. Obstructive pneumonia due to lung cancer 3. Had received antibiotics for over 72 continuous hours at the time of admission 4. Severely immunocompromised by: • Steroid treatment (prednisone ≥0.3 mg/kg/day for 3 weeks or more or ≥1 mg/kg/day for ≥7 days; if using other steroids, an equivalent dose was considered) • Treatment with cytostatics (except low doses of methotrexate: ≤15 mg/week), 5. AIDS diagnosis, lymphocyte count CD4+ <200/mm^3^ in patients over 5 years of age or less than 15 % of patients under 5 granulocytopenia <500/mm^3^, or hematologic neoplasia.	1. Hospitalization during the 2 weeks prior to recruitment 2. Obstructive pneumonia due to lung cancer 3. Severely immunocompromised by: • Steroid treatment (prednisone ≥0.3 mg/kg/day for 3 weeks or more or ≥1 mg/kg/day for ≥7 days; if using other steroids, an equivalent dose was considered) • Treatment with cytostatics (except low doses of methotrexate: ≤15 mg/week), • AIDS diagnosis, lymphocyte count CD4+ <200/mm^3^ in patients over 5 years or less than 15 % patients under 5 • granulocytopenia <500/mm^3^ or hematologic neoplasia.	1. Hospitalization during the 2 weeks prior to recruitment 2. Primary immunodeficiency or severe acquired immunodeficiency 3. Cystic fibrosis 4. Neurological alterations (cerebral palsy or neuromuscular disorders) or psychiatric alterations that kept the individual from signing the consent form, 5. Inborn errors of metabolism, 6. Bronchiolitis in children under 2 7. Hematologic neoplasia, 8. Granulocytopenia <500 cell/mm3 9. Non-infectious chronic neumopathy 10. Had AIDS or lymphocyte count CD4 < 15 % in children under 5 years 11. Individuals currently being treated with: • High-dose steroids (prednisone ≥0.3 mg/kg/day for 3 weeks or more, or ≥1 mg/kg/day for ≥7 days; if using other steroids, an equivalent dose was considered) • Treatment with cytostatics 12. Had received antibiotics for over 72 continuous hours at time of admission	1. Respiratory infections in the last month 2. Hospitalization during the 2 weeks prior to recruitment 3. Healthcare workers 4. Heart disease or chronic lung diseases 5. Cancer, granulocytopenia, or infection by HIV/AIDS 6. Individuals currently being treated with: • High-dose steroids (prednisone ≥0.3 mg/kg/day for 3 weeks or more or ≥1 mg/kg/day for ≥7 days; if using other steroids, an equivalent dose was considered) • Treatment with cytostatics • methotrexate with doses >15 mg/week 7. Had received antibiotics for over 72 continuous hours at time of admission
Sample Type	NPS	NPA stored at −80 °C	NPS and IS	NPS
Commercial PCR used	Speed-Oligo®	Speed-oligo®	Seeplex® Pneumobacter	Speed-Oligo®

### Ethics, consent and permissions

All individuals who met the inclusion criteria for the four groups signed an informed consent form in which they agreed to participate. For children, the consent form was signed by the parents or caregivers. Additionally, all children over six also signed the consent form. This study was approved by the Ethics Committee of the School of Medicine at the Universidad de Antioquia (Approval bylaws of the ethics committee: 017 of November 2011, 040 of May 2003 and 005 of May 2011) and by the Ethics Committee of participating institutions: E.S.E. Metrosalud Unidad Hospitalaria San Javier, Clínica Infantil Santa Ana, Clínica Sagrado Corazón, Clínica León XIII, Hospital Universitario San Vicente Fundación, Hospital General de Medellín, Hospital Pablo Tobón Uribe, Clínica Las Américas, Hospital San Rafael de Itagüí, Clínica CES, Hospital Marco Fidel Suárez, Clínica SOMA and Hospital Manuel Uribe Ángel.

### Clinical samples and data collection

Blood, urine, and respiratory secretion samples were taken from all patients at the time of enrollment. According to the established protocol for each study group, the NPSs of groups 1 and 4 were stored at −20 °C, whereas the NPAs of Group 2 and the NPSs and IS of group 3 were stored at −80 °C until processing. Blood samples were taken again between four and eight weeks after capture for convalescent-phase serologic testing.

### Antibodies and antigen-based detection methods

All individuals included in this study underwent the following microbiological tests for the diagnosis of atypical bacteria (following the manufacturer’s instructions):

- Detection of antibodies in acute and convalescent serum: total antibodies for *L. pneumophila* (serogroups 1 to 6, IFI Kits FOCUS Diagnostics® Cypress, CA, USA), IgM and IgG antibodies for *M. pneumoniae* (EIA Pneumobact IgM and IgG VIRCELL®, Granada, Spain), and IgG antibodies for *C. pneumoniae* (Micro-IFI IgG FOCUS Diagnostics®, Cypress, CA, USA).
- Urinary antigen for *L. Pneumophila*, serogroup 1: performed with concentrated urine (Binax NOW®, Legionella Urinary Antigen Test, Scarborough, ME, USA).

### PCR-based molecular diagnosis

Each sample was evaluated using at least two different molecular tests; one was standardized mPCR, which was performed on all samples; the second test was performed using at least one of the two commercial kits to employ a similar, standardized, and validated technique to allow comparison with mPCR. Speed-oligo® (VIRCELL, Granada, Spain) was used in the NPAs or NPSs of groups 1, 2 and 4, and Seeplex® PneumoBacter ACE detection (Seegene, Seoul, Korea) was used in the IS of Group 3 (Table 4).

For PCR testing, between 300 and 500 μl of the samples were used for DNA extraction. These respiratory samples were thawed and homogenized by vortex for 5 min, centrifuged for 10 min at 10,000 rpm, and the supernatant was discarded. DNA was extracted using the DNeasy® Blood & Tissue Kit (QIAGEN®, Hilden, Germany) and quantified using a NanoDrop® (Thermo Scientific). The total DNA volume added to the reaction was 6 μL (which we considered to be optimal after evaluating different volumes between 3 and 8 μL). The DNA concentration was not standardized. Additionally, the presence of inhibitors was ruled out amplifying the β-globin gen.

All samples were coded and processed blindly to avoid selection and information bias.

### Data analysis

For data analysis, a database was generated using Access® and was subjected to quality control prior to analysis. Statistical analyses were performed using SPSS, version 21.0. Frequency distributions were used to describe the sociodemographic and clinical characteristics of the *L. pneumophila*, *M. pneumoniae*, or *C. pneumoniae* cases identified. Sensitivity, specificity, positive and negative predictive value of mPCR, Speed-oligo®, and Seeplex® PneumoBacter were determined using the Epidat 3.1 program. Quadruplicate antibody titers and/or urinary antigen were used as a gold standard test. In addition, the concordance among the molecular techniques (mPCR, Speed-oligo® and Seeplex® PneumoBacter), between these techniques and serology, and between the different samples was evaluated using the Cohen kappa test.

## Additional files

Additional file 1:
**Nucleic acid amplification techniques and samples used for atypical bacteria detection.** (DOCX 39 kb) Additional file 2:
**The mPCR primers used for the amplification of** ***M. pneumoniae,*** ***L. pneumophila*** **and** ***C. pneumoniae.*** (DOCX 13 kb)

## Abbreviations

BAL: bronchoalveolar lavage
CAP: community-acquired pneumonia
IS: induced sputum
mPCR: multiplex PCR
NPA: nasopharyngeal aspirate
NPS: nasopharyngeal swab
