# Supplementary material for: Comparison of serological methods with PCR-based methods for the diagnosis of community-acquired pneumonia caused by atypical bacteria
Source: J Negat Results Biomed. 2016 Mar 2;15:3. doi: 10.1186/s12952-016-0047-y (PMC4774004; doi:10.1186/s12952-016-0047-y)
Supplement: Additional file 1: — Nucleic acid amplification techniques and samples used for atypical bacteria detection. (DOCX 39 kb) [file 12952_2016_47_MOESM1_ESM.docx]

**Additional file 1**. Nucleic acid amplification techniques and samples used for atypical bacteria detection

| **Author** | **Year** | **Population Type (n)** | **Detected Bacteria** | **Sample Type** | **Technique Used** | **Amplified Molecular Target** | **Most relevant results** |
| --- | --- | --- | --- | --- | --- | --- | --- |
| Amy E. Ratliff, et al. [41] | 2014 | Frozen samples M. pn (+) and (-), from adults and children with respiratory tract infections. | M. pn | 214 frozen respiratory samples (BAL, sputum, NPS, TS) | illumigene® -Loop-mediated isothermal amplification (LAMP) | Not reported | Sn: 100%, Sp: 99%, compared with culture |
| Rama Chaudhry, et al. [32] | 2013 | Children (37) and adults (97) hospitalized with CAP | M. pn | 107 TS, 19 NPA, 6 EA, and 2 BAL (frozen) | qPCR | P1 Adhesin | Sn: 34,6 % Sp: 84,2%, PPV: 34,6%, NPV: 84,2%, compared with serology |
| Bizhan Nomanpour, et al. [42] | 2012 | Patients with CAP (459) | L. pn, M. pn, C. pn and S. pn | 43 NPS, 58 sputum, and 28 BAL | Multiplex qPCR | M. pn: P1 adhesin, C. pn: *ompA*, L. pn: *mip* | Sn: 98.34%, 100% and 100% for L.pn, M.pn and C.pn. Sp: 100% for all, compared with culture |
| Fard SY, et al. [43] | 2012 | 262 patients with CAP | L. pn | Respiratory samples | qPCR | *mip* gene | Sn:100%; Sp: 96.9%, compared with culture |
| Alvaro J. Benitez, et al. [44] | 2012 | Adults (38 cases and 99 no cases), in outbreak situation | C. pn | NPS and/or OPS | qPCR | *argR* | Sn:71%, Sp: 97% |
| Mustafa MI, et al. [45] | 2011 | >15 years with CAP (46) | L. pn, M. pn and C. pn | Sputum or TS | Duplex and triplex qPCR | Not reported | L. pn: 1/46 PCR (+), serology (-); C. pn: 2/46 PCR (+), serology (+); M. pn: 3/46 serology (+) PCR (-) |
| Thurman KA, et al. [46] | 2009 | Children and adults, with CAP (97) and without CAP (166), in outbreak situation | M. pn | NPS and/or OPS | qPCR | Not reported | Sn: 9-40% Sp: >96% depending on patient age, compared with serology |
| J. A. Carrillo, et al. [47] | 2009 | Children <13 years, with CAP (54) and asymptomatic adults (55) | M. pn, L. pn, C. pn, C. bur | 54 NPS or secretion aspirates of patients with CAP, 55 TS of asymptomatic patients. | mPCR - Vircell SL kit | L. pn: *dnaJ*, M. pn: P1, C. pn: *ompA* | Calculation only for M. pn: Sn: 92.8% Sp: 100%, compared with paired serology. No asymptomatic patient was positive. Two separate PCR reactions are recommended. |
| Stephanie L, et al. | 2009 | Samples of patients with pneumonia (401) | C. pn | OPS | qPCR, “Light Upon eXtension” (LUX) and qPCR with Taqman probes | *pmp4* and *ompA* | Both tests 11/401. ompA 23/401 (12 more) |
| S. Pignanelli, et al.[14] | 2009 | Samples of patients with acute respiratory infection (50) | C. pn and M. pn | BAL | simplex and duplex qPCR | M. pn: P1 Adhesin; C. pn: *ompA* | M.pn: 1 (+) by two techniques, 1 (+) only by mqPCR. C.pn: 10/50 (+) for both tests. |
| Rachel R Higgins, et al. [13] | 2009 | Samples with suspected infection by  C. p or M. pn between 1997 and 2007 (146), previously tested by PCR for C. pn and by culture for M. pn | C. pn y M. pn | Sputum, BAL, NPA, and NPS | multiplex qPCR (Proneumo 1) | Not reported | M. pn, Sn: 95% Sp: 98% compared with culture; Sn: 100% Sp: 98% compared with Pneumobacter® (mPCR). C. pn, Sn: 91%, Sp:96% compared with homemade qPCR and Sn: 100%, Sp: 100% compared with Pneumobacter® |
| Shinobu otomo, et al. [48] | 2008 | 73 children with pneumonia | C. pn and M. pn | TS and sputum | 2 different qPCR | C. pn: MOMP gene; M. pn: 16S rRNA | C. pn, Sn: 63.6% and Sp: 100%. M. pn, Sn: 100% Sp: 100%, compared with serology |
| Karolina Gullsby, et al. [49] | 2008 | Retrospective samples of patients with (+) and (-) PCR for some bacteria (120). Prospective patients (200) | C. pn and M. pn | 120 NPS/TS retrospective and 200 prospective | Duplex qPCR | M. pn: P1 adhesin; C. pn: *ompA* | C. pn, Sn: 93% Sp: 100%. M. pn, Sn: 100% and Sp: 99%. Conventional PCR as gold standard. PCR samples (-) were all negative by duplex qPCR. |
| K. Loens, et al.[22] | 2008 | Hospitalized patients with CAP (108) and outpatient in outbreak situation (39) | L. pn, M. pn and C. pn | 251 respiratory samples (119 TS, 116 sputum, 3 gargles, 4 BAL, 4 BA, 3 PF and 2 NPA | Individual qPCR NASBA for M. pn and C. pn, and qPCR for L. pn. qPCR NASBA multiplex for all 3 bacteria | M.pn: P1, C. pn: *PstI,* L. pn: *mip* | qPCR simplex, Sn: 63,2-77,8%. qPCR NASBA, Sn: 92,1-100%. qPCR MX NASBA, Sn: 71,1-100%, The first value is compared with culture and the second with 2 amplifications. |
| Diederen BM, et al. [50] | 2008 | Adults with CAP (151) | L. pn | Sputum, EA, lung biopsies. | 2 different qPCR | 16S rRNA and *mip* | 16S rRNA Sn: 86% Sp: 95% agreement: 93%. mip Sn: 92% Sp: 98% agreement: 97%, compared with culture and/or urinary antigen |
| Yajuan Wang, et al. [51] | 2008 | Children between 1 month and 5 years with CAP (100) | L. pn, M. pn and C. pn | NPA | mPCR-Based Reverse Line Blot Hybridization (mPCR/RLB) | L. pn: *mip*, C. pn: *Pst*I, M. pn: 16S-23S rRNA region | M.pn: 2 (+) results. C. pn and L. pn: No (+) results |
| Martínez MA, et al. [21] | 2008 | >18 years with CAP (357) | M. pn | Oral lavage | Conventional PCR | 16S rRNA | Sn: 66.7%, Sp: 98.5%, PPV 78.3% and NPV: 97.3% compared with serology |
| Jonas M, et al. | 2008 | Patients with CAP (35) and controls (19), 18-35 years, in outbreak situation | M. pn | NPS and OPS | 3 different qPCR | CARDS Toxin, ATPase fragment 1 and ATPase fragment 2 | Controls: 3 (-) qPCRs.19/35 (+) qPCRs cases. The CARDS toxin has the best analytical sensitivity. |
| Diederen BM, et al.[52] | 2007 | Adults with respiratory infection without L. pn (36). Adults with infection by L.pn (68) | L. pn | 60 sera of L. pn (-) patients. 151 sera of L.pn (+) patients | 3 different qPCR | 5S rRNA, 16S rRNA and *mip* gene | 5S rRNA 37/68; mip: 36/68 (+); 16S rRNA: 21/68 (+); 20/68 (+) in all 3 PCRs. No control was (+) |
| M. Koide, et al.[53] | 2006 | 33 patients with L.pn (+) by culture, serology, acute phase, urinary antigen | L. pn | Serum | Conventional PCR | rRNA 5S | 4/15 patients urine (+), were PCR (+) |
| Miyuki Morozumi, et al.[54] | 2006 | Infants, children, adults | L. pn, M. pn, C. pn, S. py. H. in and S. pn | 365 NPA from infants and children, 14 TS from children and 40 sputum from adults | qPCR + Molecular Beacon | L. pn: *mip*; M.pn and C. pn: 16S rRNA | M. pn Sn: 100% and Sp: 95.4% compared with culture. Not evaluated for C. pn and L. pn |
| C. Ginevra, et al.[9] | 2005 | Reference strains. 30 adult patients with evidence of infection by these 3 bacteria. 220 children with respiratory infection and 86 control children. | M. pn, L. pn and C. pn | Patients: 12 EA, 7 sputum, 7 BAL, 3 NPA, and 1 NPS from adults; 220 NPA from children with respiratory infection. Controls: 86 NPA, 28 EA. | mPCR - Chlamylege kit® | C. pn: *omp*-2, M.pn: P1 adhesin; L. pn: 23S-5S rRNA | Agreement with homemade conventional PCR was 98,6% |

**TS:** throat swabs, **NPA:** nasopharyngeal aspirates, **EA:** endotracheal aspirates, **BAL:** bronchoalveolar lavage, **NPS:** nasopharyngeal swabs, **BA:** bronchial aspirate, **OPS:** oropharyngeal swabs, **Sn:** sensitivity, **Sp:** specificity, **PF:** pleural fluid
