# Supplementary material for: Comparison of serological methods with PCR-based methods for the diagnosis of community-acquired pneumonia caused by atypical bacteria
Source: J Negat Results Biomed. 2016 Mar 2;15:3. doi: 10.1186/s12952-016-0047-y (PMC4774004; doi:10.1186/s12952-016-0047-y)
Supplement: Additional file 2: — The mPCR primers used for the amplification of M. pneumoniae, L. pneumophila and C. pneumoniae. (DOCX 13 kb) [file 12952_2016_47_MOESM2_ESM.docx]

Additional file 2. The mPCR primers used for the amplification of *M. pneumoniae*, *L. pneumophila* and *C. pneumoniae*.

| Bacteria | Primers (5`- 3`) | Target gene | Fragment size |
| --- | --- | --- | --- |
| *L. pneumophila* | L.p.F: caatggctgcaaccgatgc  L.p.R: gggataacttgtgaaacctg | *mip* | 487 bp |
| *M. pneumoniae* | M.p.F: gtttgctgctaacgagtacgag  M.p.R: gtaatcatcgtctgactgcc | p1 | 360 bp |
| *C. pneumoniae* | C.p.F: cggctagaaatcaattataagactg  C.p.R: ggtgtgtttctaatacctgtcc | *Pst*I | 283 bp |

mip gene: a potentiator gene of macrophage infectivity. p1 gene: encodes P1 adhesin. PstI gene: encodes a 75 kDa protein of the heat shock protein family.
